# Characterization of Soybean Genetically Modified for Drought Tolerance in Field Conditions

**DOI:** 10.3389/fpls.2017.00448

**Published:** 2017-04-11

**Authors:** Renata Fuganti-Pagliarini, Leonardo C. Ferreira, Fabiana A. Rodrigues, Hugo B. C. Molinari, Silvana R. R. Marin, Mayla D. C. Molinari, Juliana Marcolino-Gomes, Liliane M. Mertz-Henning, José R. B. Farias, Maria C. N. de Oliveira, Norman Neumaier, Norihito Kanamori, Yasunari Fujita, Junya Mizoi, Kazuo Nakashima, Kazuko Yamaguchi-Shinozaki, Alexandre L. Nepomuceno

**Affiliations:** ^1^Embrapa Soybean, Coordination for the Improvement of Higher Education Personnel (CAPES)Londrina, Brazil; ^2^Embrapa Soybean, National Council for Scientific and Technological Development (CNPq)Londrina, Brazil; ^3^Embrapa AgroenergyBrasília, Brazil; ^4^Embrapa SoybeanLondrina, Brazil; ^5^Biological Sciences Center, Londrina State UniversityLondrina, Brazil; ^6^Japan International Research Center for Agricultural SciencesTsukuba, Japan; ^7^Laboratory of Plant Molecular Physiology, Tokyo UniversityTokyo, Japan

**Keywords:** *Glycine max*, transcription factors, transgene, ABA, water deficit, yield

## Abstract

Drought is one of the most stressful environmental factor causing yield and economic losses in many soybean-producing regions. In the last decades, transcription factors (TFs) are being used to develop genetically modified plants more tolerant to abiotic stresses. Dehydration responsive element binding (DREB) and ABA-responsive element-binding (AREB) TFs were introduced in soybean showing improved drought tolerance, under controlled conditions. However, these results may not be representative of the way in which plants behave over the entire season in the real field situation. Thus, the objectives of this study were to analyze agronomical traits and physiological parameters of *AtDREB1A* (1Ab58), *AtDREB2CA* (1Bb2193), and *AtAREB1* (1Ea2939) GM lines under irrigated (IRR) and non-irrigated (NIRR) conditions in a field experiment, over two crop seasons and quantify transgene and drought-responsive genes expression. Results from season 2013/2014 revealed that line 1Ea2939 showed higher intrinsic water use and leaf area index. Lines 1Ab58 and 1Bb2193 showed a similar behavior to wild-type plants in relation to chlorophyll content. Oil and protein contents were not affected in transgenic lines in NIRR conditions. Lodging, due to plentiful rain, impaired yield from the 1Ea2939 line in IRR conditions. qPCR results confirmed the expression of the inserted TFs and drought-responsive endogenous genes. No differences were identified in the field experiment performed in crop season 2014/2015, probably due to the optimum rainfall volume during the cycle. These field screenings showed promising results for drought tolerance. However, additional studies are needed in further crop seasons and other sites to better characterize how these plants may outperform the WT under field water deficit.

## Introduction

Drought is currently one of the most stressful environmental factor to economic crops. As a consequence, yield reductions are constant and economic and financial losses inevitable. In soybean, an important worldwide commodity, problems arising from water deficit impaired crop yield in the entire world. In Brazil, which is the second highest soybean producer worldwide and one of the few countries that could considerably increase its production in the next decades, water deficit also compromise productivity. Losses due to drought events during the period of 2003/2004 and 2014/2015 crop seasons are estimated to be in the US$46.6 billion range (Personal communication). In the crop season 2013/2014, although Brazilian production numbers increased, some regions from the South and Southeast registered significant losses (Companhia Nacional de Abastecimento [Conab], 2014).

As drought tolerance is a multigenic and quantitative trait, some difficulties arise when attempting to breed for tolerance using conventional approaches. Furthermore, time, intensity, duration and frequency of the water deficit as well as diverse plant–soil–atmosphere interactions are factors that influence plant responses (Bhatnagar-Mathur et al., 2007). As one of the strategies to cope with water deficit periods, biotechnological tools currently allow the development of genetically modified plants using gene constructs that confer drought tolerance. Among the stress-tolerant genes currently used, transcription factors (TFs) show great potential as they recognize and bind to specific DNA sequences in the regulatory regions of target genes, activating and regulating the expression of downstream genes responsible for cellular protection processes under dehydration (Shinozaki and Yamaguchi-Shinozaki, 2007).

Since existing evidence demonstrates that drought response pathways can be both abscisic acid (ABA)-independent and -dependent, TFs also acts through these two systems that govern drought-inducible gene expression. Among these TFs, dehydration responsive element binding (DREB) proteins interact with DRE/CRT by their AP2 DNA-binding domain, thus mediating downstream gene expression in the stress-responsive pathway. In contrast, the ABA-responsive element (ABRE) mainly mediates downstream gene expression in the ABA-signaling pathway (Yamaguchi-Shinozaki and Shinozaki, 2005). Insertion of the TF *AtDREB1A*, under the control of the stress-inducible rd29A promoter, successfully improved the drought tolerance responses in *Arabidopsis thaliana* (Gilmour et al., 1998; Jaglo-Ottosen et al., 1998; Liu et al., 1998), tobacco (Kasuga et al., 2004), rice (Dubouzet et al., 2003; Oh et al., 2005; Ito et al., 2006), maize (Qin et al., 2004, 2007), wheat (Pellegrineschi et al., 2004; Gao et al., 2009) and peanut (Bhatnagar-Mathur et al., 2004, 2007; Devi et al., 2011; Vadez et al., 2013). Particularly in soybean, in experiments under greenhouse conditions, transgenic lines containing TF *AtDREB1A*, presented both a higher survival rate after a severe water deficit and important physiological responses to water deprivation, such as higher stomatal conductance and the maintenance of photosynthesis and photosynthetic efficiency (Polizel et al., 2011; Rolla et al., 2013). Furthermore, data suggested that the higher survival rates of DREB plants are because of lower water use due to lower transpiration rates under well-watered conditions. In addition to physiological studies, molecular analysis revealed that drought-response genes were highly expressed in DREB1A plants subjected to severe water deficit (Polizel et al., 2011).

Another member of the DREB family, DREB2A protein, has also been used to develop genetically modified drought-tolerant plants. In *Arabidopsis*, the overexpression of a constitutively active (CA) DREB2A form resulted in significant tolerance to drought and heat stress (Sakuma et al., 2006a,b). *AtDREB2A* homologous genes were studied in maize (Qin et al., 2007), rice (Dubouzet et al., 2003), sunflower (Almogueva et al., 2009), wheat (Terashima and Takumi, 2009) and chrysanthemum (Liu et al., 2008). *AtDREB2A* was also successfully introduced in soybean. Molecular analysis conducted under hydroponic experiments showed that transgenic plants exhibited high expression of the transgene, with roots showing the highest expression levels during water deficit. Recently, Mizoi et al. (2013) identified a soybean DREB2 gene, *GmDREB2A*, and showed that its heterologous expression in *Arabidopsis* induced stress-inducible genes such as *RD29A, RD29B, HsfA3*, and *HSP70* and improved stress tolerance. These findings indicate that plants overexpressing *AtDREB2A* and *DREB2Alike* proteins have increased tolerance to abiotic stress, drought and heat, which often occur together under field conditions (Engels et al., 2013).

Considering ABA-dependent TFs, the AREB (ABA-responsive element-binding) protein family has showed interesting results conferring drought tolerance. In *Arabidopsis*, AREB acts as the major TF under abiotic stress (Kobayashi et al., 2008; Lee et al., 2010) and has been reported to regulate environmental stress responses and ABA signaling during the vegetative stage (Jakoby et al., 2002; Fujita et al., 2005; Côrrea et al., 2008; Yoshida et al., 2010). The overexpression of *AREB1* in *A. thaliana* resulted in hypersensitivity to ABA, the induction of drought-responsive genes such as *RD29B* and improved water deficit tolerance. In soybean, the *AREB1* gene was introduced and overexpressing *AtAREB1FL* lines showed the ability to survive for a period of 5 days without water under greenhouse conditions, exhibiting no leaf damage. These lines also displayed better growth and physiological performance under water-deficit (higher relative rate of shoot length, stomatal conductance, and photosynthesis) when compared to the wild type (Barbosa et al., 2012). Particularly, line 1Ea2939 showed *AtAREB1FL* expression and a greater total number of pods and seeds and increased dry matter of seeds. The best performance of line 1Ea2939 relative to BR16 plants (wild type) might be related to the mechanisms of drought prevention through reduced stomatal conductance and leaf transpiration under control conditions (no water restriction). Such results suggest that the constitutive overexpression of the TF AtAREB1 leads to an improved capacity of the soybean crop to cope with drought with no yield losses (Marinho et al., 2016).

Although all previously obtained data in greenhouses show the potential use of the TFs DREB and AREB to develop genetically modified soybean lines for drought-tolerance, these information were generated under monitored and controlled conditions of light, temperature, water, weeds, insects and diseases. According to Passioura (2012), the results obtained under controlled conditions in greenhouses may not be representative of the way in which plants behave over the entire season in the real field situation. In particular, in the vegetative stage, in the flowering-pod-filling phase, plants have a daily demand of 7–8 mm of water; thus, water deficit in these periods implies greater losses (Berlato et al., 1986; Embrapa, 2015).

Additionally, as in other countries in the world, tests in the field are a legal requirement of the Brazilian National Technical Biosafety Commission prior to approving a commercial product. Thus, it is important to test GM plants in the field to accurately gauge whether the technology is successful. As a result, considering that few studies have reported results from genetically modified crops under realistic field conditions and the fact that there is a lack of understanding with respect to the mechanisms of tolerance of DREB and AREB transgenic plants performing under a real crop season, the objectives of this study were to analyze gene expression, physiological parameters and agronomic traits of *AtDREB1A, AtDREB2CA*, and *AtAREB1FL* GM lines under irrigated and non-irrigated conditions in the reproductive stage, in a field experiment, for two crop seasons. This knowledge would provide new insights into the mechanism of drought tolerance of the DREBs and AREB plants and help soybean breeders to choose the best performing lines in real crop situations to introduce into the breeding program, aiming to develop a cultivar to be released to soybean producers.

## Materials and Methods

### Biological Material

Soybean genetically modified with *rd29A:AtDREB1A* (Patent Nos. 3183458) (line 1Ab58), *rd29A:AtDREB2CA* (Patent Nos. 3178672/PCT/JP2004/01003) (line 1Bb2193), *35S:AtAREB1FL* (Patent Nos. US-2009-0089899-A1) (line 1Ea2939) constructs and the conventional cultivar BR16 (genetic background), considered drought-sensitive [39], were sown in a field experiment, carried out during 2013/2014 and 2014/2015 crop seasons, at Embrapa Soybean (Londrina, PR, 23°18′36″S 51°09′46″O). Soil chemical corrections and cultivations were performed according to recommendations for the crop (Embrapa, 2013). All of the necessary documentation to test GM lines in field conditions were submitted and approved by The National Technical Biosafety Commission (CTNBio) (Process n° 01200.003078/2013-15 published in the Brazilian Official Journal on August 21th, 2013 by the number 3.721/2013 and Process n° 01200.003132/2014-11 published in the Brazilian Official Journal on September 09th, 2014 by the number 4.188/2014).

### Experimental Design

The experiment was carried out in the field area located in the National Soybean Research Center (23°11′ S, 51°11′ W, 630 m altitude) (Embrapa Soybean, Londrina, PR, Brazil) a branch of the Brazilian Agricultural Research Corporation during the crop seasons 2013/2014 and 2014/2015. A completely randomized split-plot design was used, with four blocks. Plots corresponded to two water conditions – irrigated (IRR, water from precipitation + irrigation when needed) and non-irrigated (NIRR, water from precipitation). Subplots corresponded to the conventional Brazilian soybean cultivar BR 16, considered drought-sensitive (Oya et al., 2004) and three transgenic isolines – 1Ab58 (*rd29A:AtDREB1A*), 1Bb2193 (*rd29A:AtDREB2CA*), 1Ea2939 (*35S:AtAREB1FL*). The area of each subplot was 220 m^2^ in IRR and NIRR conditions. Seeds were sown on November 5th, 2013 with 0.5 m spacing between rows and maintenance of 16 plants m^-1^. Cultivation conditions followed the procedures routinely adopted at Embrapa Soybean. Plants of the soybean cultivar BRS 295RR were used as a 10 m isolation border, following Brazilian legislation. Air temperature and relative air humidity were monitored daily by a weather station located close to the experimental area.

### Physiological and Agronomic Evaluations

Net CO_2_ assimilation rate (*A*), transpiration rate (*E*) and stomatal conductance (*gs*) were measured in the central leaflet of the third fully expanded trifoliate leaf (apex-to-base direction) of one plant located in the middle portion of each subplot through a portable infrared gas analyzer (LCpro-SD, ADC BioScientific) fitted for 1000 μmol m^-2^ s^-1^ photosynthetically active radiation (PAR) under sunny sky conditions between 9 and 11 a.m. (Brazilian daylight saving time). After gas exchange measurements, the instantaneous (*A*/*E*) and the intrinsic (*A*/*gs*) water use efficiency (WUE) were calculated. Chlorophyll index (SPAD) was measured in one lateral leaflet from the same above-mentioned trifoliate leaf using a portable chlorophyll meter (SPAD-502, Minolta). Then, SPAD index was converted into chlorophyll content (mg cm^-2^) through an 80% acetone standard curve (Fritschi and Ray, 2007). Plant height was the mean distance between the cotyledonary node and the stem apex from five plants per subplot. Mean length of internodes corresponded to the ratio between height per plant and number of nodes per plant. Leaf area index (LAI) corresponded to the ratio between the total leaf area, obtained through an area meter (LI-3100C, LI-COR), and the soil area occupied by the plants. Total dry matter of pods and seeds per plant and grain yield were evaluated (10 plants per subplot) in the harvest period. These measurements were carried out in all four experimental blocks, at the reproductive developmental stage.

The percentage contents of protein and oil in the samples of soybean grains at harvest were determined in whole seeds and grains using the reflectance technique of Near InfraRed (NIR) according to Heil (2010).

### Statistical Analysis

All residuals showed normal distribution and met the other assumptions of the analysis of variance (ANOVA). Thus, data were submitted to ANOVA and means compared by the Tukey’s test (*p ≤* 0.05).

### Molecular Analysis

Leaf samples from GM soybean 1Ab58, 1Bb2193, 1Ea2939 lines and the conventional cultivar BR 16 were collected from field experiments in the irrigated (IRR) and non-irrigated (NIRR) treatments. Three samples from three different blocks were individually collected, based on physiological results. Samples were immediately placed into liquid nitrogen and stored in freezer at -80°C until the moment of RNA extraction.

Total RNA was extracted from 1Ab58, 1Bb2193, 1Ea2939, and BR16 leaf samples using Trizol^®^ reagent. Following RNA extraction, samples were treated with DNAse I (Invitrogen cat n° 18047-019). To verify the presence of remaining genomic DNA, a conventional PCR was performed. cDNA synthesis was carried out using Super Script III First Strand kit (Invitrogen cat 18080-051) according to manufacturer’s instruction.

Expression level of transgenes *AtDREB1A, AtDREB2CA*, and *AtAREB1FL* was assessed using qPCR. Also, based on a search of the available literature, some genes related to drought responses were selected. The expression level of these genes was quantified under IRR and NIRR conditions. Genes related to drought response such as stomata overture/closure and osmotic adjustment, photosynthesis, metabolic and hormone pathways such as nitrogen assimilation, proteins related to drought such as dehydrins and heat shock proteins and water channels were chosen. Thus, selected genes were phosphatase *GmPP2C* (Glyma14G195200), alanine aminotransferase *GmAlaAT* (Glyma01G026700, and Glyma07G045900), Δ- 1-pyrroline-5-carboxylate synthetase - P5CS (Glyma18G034300), galactinol/Gols (Glyma10G145300), late embryogenesis abundant/LEA18 (Glyma17G164200), dehydrins (Glyma09G185500), heat shock proteins (Glyma17G072400), putative soybean aquaporin pip1/UDP galactose transporter (Glyma12G066800), putative soybean aquaporin pip2/aquaporin transporter/glycerol uptake facilitator (Glyma12G172500), ribulose-1,5-bisphosphate carboxylase/oxygenase – small chain (Glyma13G046200) and chlorophyll a/b binding protein – Cab21 (Glyma16G165800).

Using gene sequences obtained from Phytozome, sets of primers for each gene were designed using Primer3Plus platform available online ^1^ (**Additional File S1**). To verify homo- and heterodimers and hairpin formation, the Multiple primer analyzer software was used ^2^. Forward and reverse primers and amplified fragment size are described in the **Additional File S1**. PCR reactions were carried out in biological and technical triplicate using the kit Platinum^®^ SYBR Green^®^ qPCRSuperMix-UDG with ROX according to the manufacturer’s instructions in a 7900HT Fast Real-Time PCR System with 384-Well Block (Applied Biosystems cat n° 4329001). The β-actin gene (No Access: GMU60500) was used as the reference gene (Stolf-Moreira et al., 2011).

The efficiency of the amplification reaction was estimated using five serial dilutions of cDNA (1, 5, 25, 125, and 625×). To compute the efficiency of the reaction, the equation: *E* = [10-1/slope]-1 was applied, in which only primers with efficiency above 90% were used. The cycling parameters for the reactions were 50°C for 2 min and 95°C for 10 min, followed by 40 cycles of 95°C for 15 s and 60°C for 1 min. To check the specificity of the amplified products, a dissociation curve was generated after the end of each reaction. The relative expression was determined by normalization to the reference gene β-actin. The expression was calculated by the 2^-ΔΔCt^ method (Bustin, 2002).

An *in silico* research for putative *cis*-elements was also performed using gene sequences obtained from Phytozome and software Genomatix^3^ aiming to identify possible TF sites in the promoter regions and also other sites related to drought tolerance mechanisms.

## Results

### Physiological and Agronomical Evaluations

**FIGURE 1:**
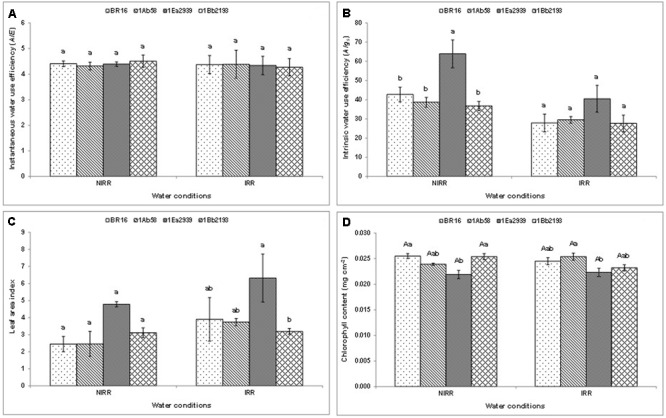
**Instantaneous (A)**, intrinsic **(B)** water use efficiency (WUE), leaf area index (LAI) **(C)** and chlorophyll content **(D)** of the transgenic lines 1Ab58, 1Ea2939, and 1Bb2193, and WT plants (BR 16 cultivar) subjected to non-irrigated (NIRR) and irrigated (IRR) treatments under field conditions. For **(A–C)**, in each water condition, means ± standard error followed by the same letter did not differ according to the Tukey’s test (*p* ≤ 0.05). For **(D)**, means ± standard error followed by the same uppercase letters (between water conditions) and lowercase letters (among plant materials) did not differ according to the Tukey’s test (*p* ≤ 0.05). *n* = 4 blocks.

Results from crop season 2013/2014, for instantaneous (*A*/*E*) and intrinsic (*A*/*gs*) water use efficiency, and LAI (**Figures 1A–C**) did not show any significant interaction between water conditions and plant materials. In each water condition, there were no differences between plant materials regarding *A*/*E* (**Figure 1A**). However, the line 1Ea2939 showed higher *A*/*gs* than the other plant materials under NIRR treatment (**Figure 1B**). Furthermore, in each water condition, the line 1Ea2939 presented higher values for LAI than those of other plant materials in general. The lines 1Ab58 and 1Bb2193 showed a similar behavior than WT plants in relation to chlorophyll content (**Figure 1D**) regardless of water condition. Conversely, the line 1Ea2939 had lower chlorophyll content relative to WT plants under NIRR conditions (**Figure 1D**).

**FIGURE 2:**
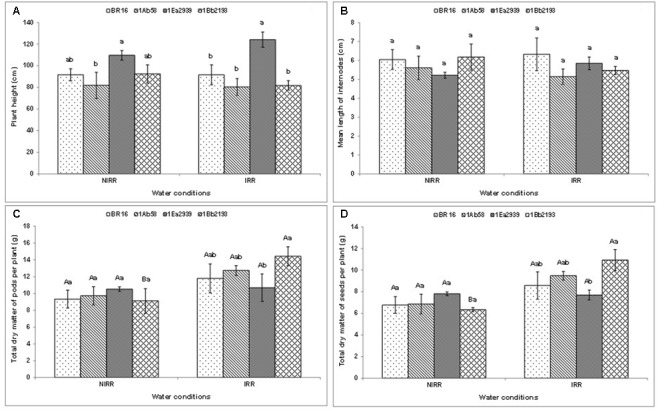
**Plant height (A)**, mean length of internodes **(B)**, and total dry matter of pods **(C)** and seeds **(D)** per plant of the transgenic lines 1Ab58, 1Ea2939, and 1Bb2193, and WT plants (BR 16 cultivar) subjected to non-irrigated (NIRR) and irrigated (IRR) treatments under field conditions. For **(A,B)**, in each water condition, means ± standard error followed by the same letter did not differ according to the Tukey’s test (*p* ≤ 0.05). For **(C,D)**, means ± standard error followed by the same uppercase letters (between water conditions) and lowercase letters (among plant materials) did not differ according to the Tukey’s test (*p* ≤ 0.05). *n* = 4 blocks.

With regard to plant height (**Figure 2A**) and the mean length of internodes (**Figure 2B**), there was no significant interaction between water conditions and plant materials. Thus, in each water condition, the line 1Ea2939 presented higher values for plant height than those of other plant materials in general. Furthermore, in both agronomic traits, the lines 1Ab58 and 1Bb2193 showed similar values relative to those of WT genotype under IRR and NIRR treatments. Plant materials did not show differences as to mean length of internodes in each condition (**Figure 2B**). Moreover, plants showed similar results between IRR and NIRR treatments regarding total dry matter of pods and seeds per plant, except for the line 1Bb2193, which showed lower values under NIRR for both agronomical traits (**Figures 2C,D**). In both traits, transgenic lines showed similar results to those of WT plants (BR 16 cultivar) regardless of water conditions. Furthermore, the line 1Ea2939 had lower values of both traits than those of the line 1Bb2193 under IRR treatment.

**FIGURE 3:**
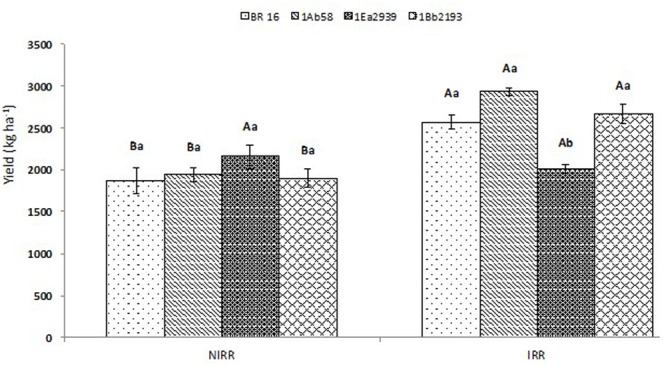
**Yield of the transgenic lines 1Ab58, 1Ea2939, and 1Bb2193, and WT plants (BR 16 cultivar) subjected to non-irrigated (NIRR) and irrigated (IRR) treatments under field conditions.** Means ± standard error followed by the same uppercase letters (between water conditions) and lowercase letters (among plant materials) did not differ according to the Tukey’s test (*p* ≤ 0.05). *n* = 4 blocks.

Yield results showed significant interactions between water conditions and plant materials (**Figure 3**). No differences were identified between transgenic lines and WT plants at NIRR conditions; however, line 1Ea2939 presented the highest yield value reaching 2.153 kg ha^-1^, showing no differences with the final yield obtained for this line at IRR treatment (2.012 kg ha^-1^). Nevertheless, in this water condition, line 1Ea2939 presented lower yield values when compared to other transgenic lines and WT plants (BR 16 cultivar), due to a severe lodging that occurred after a plentiful rain (341.4 mm), decreasing productivity and final potential yield numbers (**Additional Files S2**, **S3**). Before the abundant rain, line 1Ea2939 showed a higher number of nodes (five plants average) and higher number of pods per plant, thus being more impaired in the harvest due to lodging (**Additional File S4**).

**FIGURE 4:**
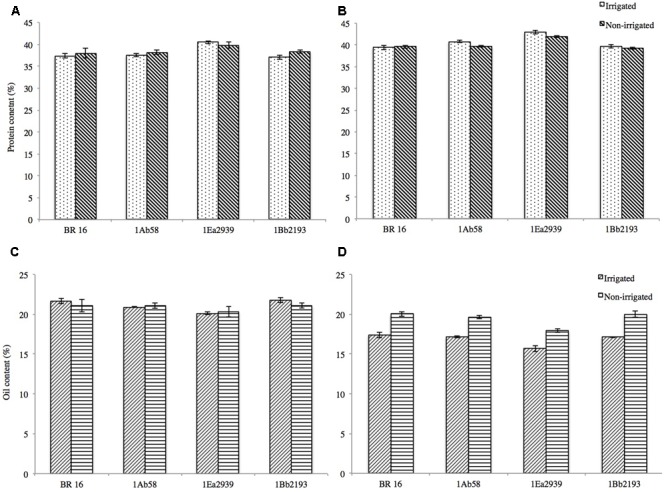
**Protein (%) and oil (%) content in soybean GM lines 1Ab58, 1Bb2193, and 1Ea2939 and WT plants (BR 16 cultivar) subjected to irrigated (IRR) and non-irrigated (NIRR) treatments under field conditions. (A,C)** Data from crop season 2013/2014. **(B,D)** Data from crop season 2014/2015. Values represent mean ± standard error; *n* = 4 replicates.

Protein and oil contents in soybean seeds were not affected by insertion of the FTs DREB1A, DREB2A, and AREB1 (**Figures 4A–D**). In crop season 2013/2014, for protein content under IRR conditions, final values ranged from 37.07% (1Bb2193) to 40.50% (1Ea2939) and for NIRR, values varied from 38.01% (BR 16 cultivar) to 39.77% (1Ea2939) (**Figure 4A**). Oil content in the seeds ranged from 20.11% (1Ea2939) to 21.76% (1Bb2193) for IRR conditions and from 20.31% (BR 16 cultivar) to 21.08% (1Bb2193) for the NIRR treatment (**Figure 4C**). In crop season 2014/2015, overall, values for protein content were higher and for oil content lower when compared to crop season 2013/2014, with line 1Ea2939 reaching values for protein content of around 41–42% and between 15 and 17% for oil content (**Figures 4B,D**). However, it must be emphasized that, in both crop seasons, line 1Ea2939 showed the highest protein content and the lowest oil content, both in IRR and NIRR conditions, relative to the other plant materials.

No differences were identified for physiological and agronomic parameters in the field experiment performed in crop season 2014/2015, probably due to the optimum rainfall volume during the whole cycle, thus resulting in scarce water stress in plants. According to the data collected by the weather station located in the experiment spot, a total rainfall of 790.8 mm was registered (**Additional File S5**). The recommendations for soybean crop for water requirements vary between 450 and 800 mm/cycle, depending on weather conditions, crop management and cycle duration (Embrapa, 2013). Although a short period of water deficit occurred in October/2014, the experiment was sown on November 6th. Thus, as no significant water deficit period occurred during the cycle, no differences were shown between GM lines and BR16 cultivar. As no differences were identified, no molecular analyses were performed.

### Molecular Analysis

Gene expression analysis, performed in samples collected in crop season 2013/2014 showed that transgenes *AtDREB1A, AtDREB2CA*, and *AtAREB1FL* were induced under NIRR conditions in each respective transgenic line. Among TFs, the higher expression was identified for *AtDREB1A* gene (line 1Ab58) with expression value reaching 4.806x. Transgenes *AtDREB2CA* and *AtAREB1FL* gene expression were 2.91x and 1.34x. No expression was identified for BR16 soybean conventional cultivar.

**FIGURE 5:**
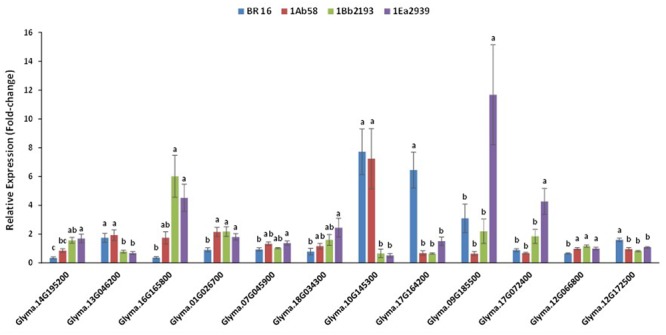
**Relative expression level of phosphatase *GmPP2C* (Glyma14g195200), ribulose-1,5-bisphosphate carboxylase/oxygenase – small chain (Glyma13g046200), chlorophyll a/b binding protein – Cab21 (Glyma16g165800), alanine aminotransferase *GmAlaAT* (Glyma01g026700 and Glyma07g045900), Δ- 1-pyrroline-5-carboxylate synthetase – P5CS (Glyma18g034300), galactinol/Gols (Glyma10g145300), late embryogenesis abundant/LEA18 (Glyma17g164200), dehydrins (Glyma09g185500), heat shock proteins (Glyma17g072400), putative soybean aquaporin pip1/UDP galactose transporter (Glyma12g066800), putative soybean aquaporin pip2/aquaporin transporter/glycerol uptake facilitator (Glyma12g172500) genes, in plants of transgenic lines 1Ab58, 1Bb2193, and 1EA2939 and WT plants (BR 16 cultivar) subjected to non-irrigated (NIRR) and irrigated (IRR) treatments under field conditions.** Expression was normalized with the reference gene Gmβ-actin. On each gene, means ± standard error followed by the same letter do not differ according to the Tukey test (*p* ≤ 0.05); *n* = 9.

All of the analyzed endogenous drought-responsive genes showed statistical differences between plant materials, although pattern expression varied depending on the GM line. However, some expression behaviors, regardless of being up or down, were identified (**Figure 5**).

Thus, for Glyma01G026700 (alanine aminotransferase *GmAlaAT*), Glyma17G164200 (late embryogenesis abundant – LEA), Glyma12G066800 (putative soybean aquaporin pip1/UDP galactose transporter) and Glyma12G172500 (putative soybean aquaporin pip2/aquaporin transporter/glycerol uptake facilitator), no statistical difference was found between GM lines, but they were different from BR 16 (WT plants). For Glyma13G046200 (ribulose-1,5-bisphosphate carboxylase/oxygenase-small chain) and Glyma10G145300 (galactinol), GM lines 1Ea2939 and 1Bb2193 showed similar expression to each other, but different expression to that of BR 16 and 1Ab58. Furthermore, the GM line 1Ea2939 presented higher expression than other plant materials for Glyma09g185500 (dehydrins) and Glyma17G072400 (heat shock protein) (**Figure 5**).

For the phosphatase *GmPP2C* (Glyma14G195200) gene, the GM lines 1Ea2939 and 1Bb2193 showed higher expression than 1Ab58 and the conventional cultivar BR 16. However, 1Ab58 and 1Bb2193 also shared similar expression profile. Considering Glyma13G046200, GM lines 1Ea2939 and 1Bb2193 showed similar expression each other, but lower expression than BR 16 and the line 1Ab58. For chlorophyll a/b binding protein – Cab21 (Glyma16G165800) gene, the GM lines 1Ea2939 and 1Bb2193 showed similar expression to each other, and higher expression than BR 16 and 1Ab58, which also presented expression that was strongly similar to the conventional cultivar. For genes involved in the nitrogen assimilation process, results showed that no significant difference was identified between transgenic lines (Glyma01G026700), although lines 1Ab58 and 1Bb2193 were statistically similar to BR16 for Glyma07G045900. However, for both genes, the AREB1 line (1Ea2939) showed different expression when compared to the conventional cultivar. This expression pattern was also found for the Δ-1-pyrroline-5-carboxylate synthetase (P5CS) gene (Glyma18G034300) (**Figure 5**).

Considering genes involved in osmotic adjustment, such as galactinol/Gols (Glyma10G145300), higher expression was detected for the GM line 1Ab58 and BR 16 plants. For the LEA protein gene (Glyma17G164200), no significant difference was identified between transgenic lines; however, they showed lower expression than BR16 plants. For the dehydrin gene (Glyma09G185500) and heat shock protein gene (Glyma17G072400), the GM line 1Ea2939 showed higher expression than the other plant materials. Water channel-related genes such as Glyma12G066800 and Glyma12G172500 showed similar expression between GM lines, but their expression was higher and lower than that of the conventional cultivar BR16, respectively (**Figure 5**).

Besides standard plant promoters motifs such as the TATA-box and CCAAT-box and the DRE (conserved motif sequence A/G)CCGACNT) and ABRE (conserved motif sequence PyACGTGG/TC) *cis*-elements, which are specific binding sites for DREB and AREB TFs, other putative *cis*-elements related to drought response mechanisms in plants were identified in the promoter regions of the endogenous genes analyzed. Circadian cycle control and light response element motifs such as evening element, circadian clock associated 1, late elongated hypocotyl, GAP-box, and time-of-day-specific elements were identified. Motifs related to sugar-responsive genes and heat shock responses were also present in the genes’ promoter regions (**Additional File 6**).

## Discussion

As the world’s weather is altering, probably due to climate change, the development of drought-tolerant crops is gaining prominence. In general, plant drought resistance involves four major mechanisms: drought avoidance (DA), drought tolerance (DT), drought escape (DE), and drought recovery. DA is mainly characterized by the maintenance of high plant water potentials in the presence of water shortage, and is accomplished through three basic general strategies: (1) reducing water loss via rapid stomatal closure, leaf rolling and increasing wax accumulation on the leaf surface; (2) enhancing the water uptake ability through a well-developed root system and enhancing the water storage abilities in specific organs; and (3) accelerating or decelerating the conversion from vegetative to reproductive growth to avoid complete abortion at the severe drought stress stage. DT refers to the ability of plants to sustain a certain level of physiological activities under severe drought conditions through the regulation of thousands of genes and series of metabolic pathways to reduce or repair the resulting stress damage (Fang and Xiong, 2015). The different expression pattern identified for endogenous soybeans genes related to drought response (**Figure 5**) illustrates how one or more of these mechanisms can be activated and interact within and between them to cope with water deficit periods.

The strategy to improve drought tolerance by inserting TFs that regulate the expression of several drought-responsive genes, from either the ABA (ABA)-independent or -dependent response pathway has already shown promising results for model plants such as *Arabidopsis* (Gilmour et al., 1998; Jaglo-Ottosen et al., 1998; Liu et al., 1998; Kasuga et al., 1999; Fujita et al., 2005), as well as for important economic crops like potato (Behnam et al., 2006), tobacco (Kasuga et al., 2004), rice (Dubouzet et al., 2003; Oh et al., 2005; Ito et al., 2006), wheat (Pellegrineschi et al., 2004; Gao et al., 2009), maize (Qin et al., 2004, 2007), peanut (Bhatnagar-Mathur et al., 2007; Devi et al., 2011; Vadez et al., 2013), and soybean (Polizel et al., 2011; Barbosa et al., 2012; Rolla et al., 2013; Leite et al., 2014; Marinho et al., 2016).

Using soybean lines GM with FTs DREB and AREB, most of the previous molecular and physiological characterizations were performed in greenhouses under controlled conditions of light, temperature, water, weeds, insects, and diseases. In this contained environment, results for “concept proof” were promising (Polizel et al., 2011; Barbosa et al., 2012; Rolla et al., 2013; Leite et al., 2014; Marinho et al., 2016); however, when the main objective is a commercial cultivar release, real field condition screenings are necessary, as, according to Passioura (2012), the results obtained under controlled conditions in greenhouses may not be representative of the way in which plants behave over the entire season in the real field situation. Still, according to our observations, in containment conditions, plants are not able to express their total potential, as limitations due to pot size and controlled water amount, temperature fluctuations, diseases and pests do not challenge the organism as a whole, but reduce environmental real situations.

Under drought, photosynthesis is among the primary processes which are down-regulated. Molecularly, in response to the stress condition, mRNA levels of the light and dark reaction genes (such as *RbcS*, Glyma13G046200, in lines 1Bb2193 and 1Ea2939, **Figure 5**) rapidly reduced, a phenomenon referred to as stress-induced mRNA decay (SMD) (Park et al., 2012). Physiologically, as observed, in NIRR conditions line 1Ea2939 decreased gas exchanges (data not presented), by stomatal closure (lower *gs*) which probably occurs as a mechanism to keep water in the cell, which, however, did not imply losses in the use of the free CO_2_, as instantaneous water use efficiency (*A*/*E*) was equal to that of the other transgenic lines and WT plants and intrinsic water use efficiency (*A*/*g_s_*) was higher (**Figure 1**). The explanation for stomatal closure during water stress in seed plants relies on the phytohormone ABA, which is seen as a cornerstone of stomatal function, because it has been shown to trigger responses in guard cell membrane channels and transporters that cause a reduction in guard cell turgor, thereby closing stomata (Bauer et al., 2013). In the field, few studies show a strong correlation between the level of ABA and *gs* during water deficit. In field-grown grapevine (*Vitis vinifera* L. cv Cabernet Sauvignon), a correlation between ABA abundance in the xylem sap and *gs* strongly supported the involvement of ABA in stomatal regulation under field conditions. The different irrigation levels significantly altered the Ψ_leaf_ and *gs* of the vines across two crop seasons (Speirs et al., 2013).

In the ABA-mediated stomatal closure, the light-harvesting chlorophyll a/b-binding (LHCB) proteins can also be involved (Xu et al., 2012). In the present study, Glyma16G165800, a LHCB protein was up-regulated in lines 1Bb2193 and 1Ea2939 (**Figure 5**) and ABRE *cis*-elements were found in the promoter region (**Additional File S6**). In higher plants, the superfamily of these proteins is composed of more than 20 members associated with photosystem I (PSI) or photosystem II (PSII). As observed here for soybean, in *Arabidopsis*, LHCBs positively regulate plant drought tolerance by functioning to positively control stomatal movement, through guard cell signaling, in response to ABA (Xu et al., 2012). In date palm (*Phoenix dactylifera* L.) cultivar “Sagie” subjected to drought, LHCBs were up-regulated. The accumulation of these proteins in PSI exposed to salt and drought stress might represent one of the strategies to prevent or lower light stress-induced damage. It was proposed that these proteins might have a protective function within PSII under stress conditions, either by binding free chlorophyll molecules and preventing the formation of free radicals and/or by acting as sinks for excitation energy, because under stress conditions, a mobile pool of these proteins moves from PSII to PSI due to the reversible phosphorylation of these proteins by a thylakoid bound kinase (El Rabey et al., 2015).

Furthermore, in response to water deficit, ABA binds directly to the PYR/PYL/RCAR family of ABA receptors, thus resulting in the inhibition of type-C protein phosphatases 2C (PP2C). In the absence of protein phosphatase PP2C activity, SnRK2 protein kinases are free to be activated by autophosphorylation, and activate downstream target genes as a result (Klingler et al., 2010; Boneh et al., 2012). In the cytoplasm, kinases may also phosphorylate anionic slow channels (SLAC1) or potassium channels (kat1) to induce stomatal closure in response to ABA (Umezawa et al., 2010).

Thus, stomata closure exhibited by *AtAREB1FL* line 1Ea2939 was probably triggered by the combination of different physiological (**Figures 1**, **2**) and molecular mechanisms, as Glyma14G195200 (phosphatase *GmPP2C*) was up-regulated in this line (but also in line 1Bb2193) (**Figure 5**).

Considering the obtained molecular and physiological data together, it suggests that GMs lines 1Bb2193 and 1Ea2939 share some similarities in the drought response behavior, targeting more than one mechanism to cope with water deficit periods combining modulation of the gene expression profile and physiological responses, as a strategy to conserve more water and protect cells during water starvation.

However, since drought response is a complex mechanism and plants, as sessile organisms, cannot avoid abiotic stresses by sheltering, they have evolved a series of mechanisms that alone or combined together overcome drought conditions. Thus, many other molecules are synthesized to cope with water deficit. Considering alanine aminotransferase (AlaAT) (Glyma01G026700 and Glyma07G045900) genes, which are evolved in nitrogen metabolism, no differences were identified among the GM lines, which presented slight up-regulation when compared to BR16. This expression profile also occurs for proline (P5CS – Glyma18G034300). In general, AlaAT plays a key role in plant metabolism by linking primary carbon metabolism with the synthesis of amino acids. If considered that under drought, a decrease in protein synthesis, protein levels and activity of enzymes occurs, thus, in GM lines, AlaATs might be ensuring amino acid and protein synthesis during drought, which explains a higher proline accumulation in transgenic lines when compared to BR16. This amino acid, under stress conditions, as well as acting as an excellent osmolyte, plays three major roles, as a metal chelator, an antioxidative defense molecule and a signaling molecule. Overproduction of proline in plants imparts stress tolerance by maintaining cell turgor or osmotic balance; stabilizing membranes to prevent electrolyte leakage; and bringing concentrations of reactive oxygen species (ROS) within normal ranges, thus preventing oxidative burst in plants (Hayat et al., 2012). In wheat, drought stress tolerance at a cellular level was associated with the ability to accumulate proline and high water level conservation (Sultan et al., 2012). In sugarcane, proline accumulation in transgenic plants under water-deficit stress acts in the cytoplasmatic osmotic adjustment but also as a component of the antioxidative defense system (Molinari et al., 2007). In soybean, proline content increased in the leaves and nodules of plants subjected to water deficit during the flowering stage (Silvente et al., 2012). Also, a positive and significant correlation among the activity of antioxidant enzymes, ABA and proline content with seed and oil yield in water deficit stress was observed (Masoumi et al., 2011). Higher accumulation of proline was still associated with less injury and a greater amount of water retention in a stress-tolerant soybean genotype (Angra et al., 2010).

Dehydrins (DHNs are the group II, D11 family of LEAs – Late Embryogenesis Abundant) (Glyma09G185500) and heat shock proteins (HSPs) (Glyma17G072400) were also induced under drought treatment, in line *AtAREB1FL* 1Ea2939, which presented the higher expression among transgenic and BR16 plants (**Figure 5**). Dehydrins are exclusively found in plants and accumulate in the late stages of embryogenesis, when water content in seeds declines, or in response to various stressors. The presence of these proteins has been observed in several independent studies on drought and salinity stresses as well as on cold acclimation and after ABA treatment (Rorat et al., 2006; Tripepi et al., 2011). DHNs play critical roles in desiccation tolerance by capturing water, stabilizing and protecting the structure and function of proteins and membranes, as well as acting as molecular chaperons (as heat shock proteins) and hydrophilic solutes to protect cells from the damage of water stress (Hand et al., 2011). In *Olea europaea* L. Subsp. *europaea*, var. *sylvestris*, the wild plant of olive, the expression *OesDHN* was induced under mild drought stress. In addition, *Arabidopsis* transgenic plants showed a better tolerance to osmotic stress suggesting that *OesDHN* expression is induced by drought stress and is able to confer osmotic stress tolerance (Chiappetta et al., 2015). In grapevine, *DHN1* and *DHN2* genes were induced by drought, cold, heat, embryogenesis, as well as the application of ABA, salicylic acid (SA), and methyl jasmonate (MeJA) and a higher number of putative ABRE *cis*-elements was located in *DHN1* and *DHN2* promoters (Yang et al., 2012), which were also identified here in soybean (Glyma09G185500, **Additional File S5**).

It has also been suggested that some dehydrins probably play a role in antioxidative defense response directly by their radical scavenging activity (Hara et al., 2004), or indirectly by their capability of binding toxic metals and preventing the production of ROS (Hara et al., 2005). There are likewise some indications about the relationship between the response to oxidative stress and the response to heat stress, as both stresses induce the pathways leading to the expression/accumulation of Hsps (Al-Whaibi, 2011). Thus, dehydrin and heat shock proteins might be acting combined in line 1Ea2939 to protect cells against drought but also with potential to guard from other different stresses. The expression of other drought-responsive molecules suggests that line 1Ea2939 displays yet more mechanisms to cope with water deficit periods, keeping water in the cell to maintain plant metabolism aiming to sustain yield.

As observed here, lines 1Bb2193 and 1Ea2939, containing ABA-independent and -dependent TFs (DREB2A and AREB1), respectively, showed a similar expression pattern under drought conditions for some endogenous genes (**Figure 5**). Interestingly, increasing evidence shows that DRE/CRT can act as a coupling element of the ABRE *cis*-element to regulate downstream gene expression (Narusaka et al., 2003). Thus, there exists a comprehensive connection between stress-responsive and ABA-signaling pathways (Shinozaki and Yamaguchi-Shinozaki, 2000; Shinozaki et al., 2003). A few of the DREB-type TFs were found to be involved in the ABA-dependent pathway (Egawa et al., 2006). In maize, ZmABI4, a DREB protein that shows ABA-induced expression, binds to CE1 and acts as a coupling element of the ABRE (Niu et al., 2002). Consistently, overexpression of DREB1D/CBF4, which is an ABA-responsive gene of *Arabidopsis*, activates the expression of drought and cold-related downstream genes that contain the DRE/CRT *cis*-element (Knight et al., 2004). In rice, three ABRE elements were identified in the promoter region of *ARAG1* gene, which encodes a DREB-like protein containing the characterized AP2 DNA binding domain (Zhao et al., 2010). In *Arabidopsis*, evidence shows that AREB/ABF proteins physically interact with DREB/CBFs including DREB1A, DREB2A, and DREB2C (Lee et al., 2010). In *Setaria italica*, the transcript level of *SiARDP*, an abscisic-responsive DREB-binding protein, increased not only after drought, high salt and low temperature stresses, but also after an ABA treatment in foxtail millet seedlings (Li et al., 2014). Furthermore, transient-expression analyses coupled with ChIP (Chromatin Immunoprecipitation) assays have shown AREB/ABFs such as AREB1, AREB2, and ABF3 can bind to the promoter of DREB2A and thereby induce them in an ABRE-dependent manner (Kim et al., 2011).

As a drought-sensitive genotype, WT BR16 also targets molecular mechanisms in response to water deficit. Thus, expression profile showed that LEA protein (Glyma17G164200) was highly induced (**Figure 5**). In the conventional cultivar, this protein, acting as molecular chaperone, might be associated with the osmotic adjustment (OA) mechanism, as galactinol (*Gols* – Glyma10G145300) and aquaporin (Glyma12G172500) also presented higher expression levels. In the OA, the accumulation of a variety of organic and inorganic substances increases the concentration in the cytochylema, reducing the osmotic potential and improving cell water retention in response to water stress. In overexpressing *Arabidopsis*, a wheat (*Triticum durum*) group 2 LEA protein (DHN-5) improved tolerance to salt and drought stress through osmotic adjustment. The water potential was more negative in transgenic than in wild type plants and, in addition, these plants have lower water loss rate under water stress (Brini et al., 2007). In *Arabidopsis*, sugar analysis showed that drought, high salinity and cold treated plants accumulate a large amount of raffinose and galactinol, functioning as osmoprotectants in drought-stress tolerance (Taji et al., 2002). In poplar (*Populus* spp.), the osmolytes raffinose and galactinol exhibited increased abundance under drought stress (Barchet et al., 2013). Also, in *Coffea arabica* and *C. canephora, GolS* genes were highly induced under water deficit periods (Dos Santos et al., 2011, 2015). It must be emphasized that, although BR16 (WT) plants seemed to have presented OA mechanism under drought stress, such a response was not more efficient than the mechanisms shown by the transgenic lines, based on physiological, growth and agronomical results (**Figures 1**–**3**).

Besides these molecules, aquaporins (AQP), which were differentially expressed in BR16 (Glyma12G066800 and Gyma12G172500) (**Figure 5**), have also been demonstrated to have crucial roles in OA in plant cells. AQPs participate in the rapid transmembrane water flow and when plants are subjected to drought or salt conditions, increased transport of water across membranes is crucial to maintain a healthy physiological status. In banana (*Musa acuminata* L.), the aquaporin gene *MaPIP1;1* was induced in leaves and roots after salinity stress and simulated drought treatments, and overexpressing *Arabidopsis* displayed better growth, more green leaves, higher survival rates and lower water loss rate compared to WT under drought conditions. Transgenic lines also experimented less lipid peroxidation and membrane injury, and improved osmotic adjustment under drought treatment (Xu et al., 2014). Once more, these data suggest that soybean conventional cultivar BR16 might be targeting, mainly, the OA mechanism to cope with water deficit, as GM line 1Ab58.

Finally, when yield parameters were assessed in crop season 2013/2014, results showed lower productivity for 1Ea2939 plants under IRR conditions (2.0212 kg ha^-1^) when compared to NIRR (2.153 kg ha^-1^). This difference of approximately 140 kg ha^-1^ was due to a lodging that occurred in the IRR treatment (**Additional File S2**). As observed in the climatologic water balance (**Additional File S3**), after a severe drought, plentiful rain occurred (341.4 mm) in a short period. Since 1Ea2939 plants were higher (**Figure 2A**), exhibiting greater LAI (**Figure 1C**), and a higher number of nodes and total pod number per plant (**Additional File S4**), lodging implications were more austere for this GM line, decreasing the final production in this experimental plot. However, based on the number of nodes and number of pods per plant on 17th February 2014 (after water deficit and before rainfall – **Additional File S4**), we can consider that the potential productivity for this line is presumably greater. Line 1Ea2939 showed 21 nodes/plant (average of five plants) in both IRR and NIRR conditions, while other transgenic lines and WT plants presented an average ranging from 14 to 15 nodes in both treatments. A positive relationship between pods, nodes and yield or nodes and pods and seeds has been reported (Kahlon et al., 2011; Egli, 2013). This association is also related to environmental conditions and other plant characteristics such as photosynthesis, crop growth rate and maturity, since later maturing cultivars have longer vegetative growth periods and more nodes than earlier cultivars (88). Line 1Ea2939 showed longer cycle (150 and 153 days in IRR and NIRR, respectively) than other transgenic lines and WT plants (ranging from 128 and 132 days in both water conditions). In general, data demonstrated that this line was able to cope with drought through some plant defense mechanisms and invest in growth and productivity, in association with a higher intrinsic WUE (*A*/*gs*) (**Figure 1B**).

Besides increased yields, soybean producers and agricultural biotechnology worldwide are searching for grain quality to benefit health, growth and/or nutrition of animals, as this grain remains the most important and preferred source of high quality vegetable protein for animal feed manufacture. The chemical composition of soybeans may vary somewhat according to variety (genetic component) and growing conditions (environmental component). Through plant breeding, it has been possible to obtain protein levels between 40 and 45%, and lipid levels between 18 and 20%. Overall, GM lines presented protein and oil content values accepted by crushing industry, meting quality references, and commercial specifications (Embrapa, 2015). The maintenance of these parameters is essential to be considered in the development of GM lines, as it adds value to the grain and ensures the competitiveness of soy in the world market. Transgenic DREB and AREB lines evaluated here under water deficit conditions presented acceptable oil and protein percentages, enabling cultivar obtained from these lines to enter in feed market for poultry, pork, cattle, other farm animals and pets.

## Conclusion

Transcription factors introduced in soybean conventional cultivar BR16 and endogenous genes related to water deficit-responses showed higher expression in drought condition, showing that plants can modulate the metabolism in response to this adverse environmental circumstance by targeting different mechanisms, aiming to survival and keep productivity. Although these soybean transgenic lines expressing TFs DREB in general did not present a better performance when compared to WT plants (BR16 conventional cultivar), in field conditions under water deficit condition, a better performance was observed for 1Ea2939 AREB line which showed a higher performance than WT and other GM lines. Oil and protein content in the transgenic lines were not affected by the introduction of TFs, ensuring possible commercial cultivars derived form these lines competitiveness in livestock market.

## Author Contributions

RF-P and LF contributed equally for the manuscript, being responsible for experiments installation, data collect and analysis, and manuscript write. FR helped in molecular analysis and manuscript review. HM helped with manuscript writing and editing. SM helped in the laboratory procedures and MM was involved with experiments performed in the greenhouse. JM-G and LM-H helped with manuscript review. JF and NN gave important support on physiological data analysis and MdO performed all statistical analysis. NK, YF, and JM developed the genetic construction used to obtain GM soybean lines. KN and KY-S participated in the experimental and molecular characterization design and reviewed the manuscript. AN is the coordinator of the research group and was directly involved in all stages of the research described in this manuscript.

## Conflict of Interest Statement

The authors declare that the research was conducted in the absence of any commercial or financial relationships that could be construed as a potential conflict of interest.

## Footnotes

1 http://primer3plus.com/cgi-bin/dev/primer3plus.cgi

2 http://www.thermoscientificbio.com/webtools/multipleprimer/

3 http://www.genomatix.de

## Supplementary Material

FILE S1 **Genes, Glymas, primers sequences, and fragment sizes of all endogenous genes analyzed**.

FILE S2 **Photo from lodging and pod details from 1Ea2939 line. Lodging occurred after plentiful rain (341.4 mm) in a short period**.

FILE S3 **Climatologic water balance from crop season 2013/2014, showing rainfall (mm), water deficit, water withdrawal and maximum temperature (°C).** Graphic is scaled in a 10-day period from October 2013 to April 2014.

FILE S4 **Number of nodes and total pod number per plant for GM lines 1Ab58, 1Ea2939, and 1Bb2193 and WT plants (BR 16 cultivar) on February 17th, 2014 and on harvest (April, 2014), in non-irrigated (NIRR) and irrigated (IRR) treatments under field conditions**.

FILE S5 **Climatologic water balance from crop season 2014/2015, showing rainfall (mm), deficit, and water withdrawal, scaled in a 10-day period from October 2014 to April 2015**.

FILE S6 **Putative *cis*-elements present in the positive strand of the endogenous genes analyzed**.
