# Supplementary figures and images for: Characterization of Soybean Genetically Modified for Drought Tolerance in Field Conditions
Source: Front Plant Sci. 2017 Apr 11;8:448. doi: 10.3389/fpls.2017.00448 (PMC5387084; doi:10.3389/fpls.2017.00448)

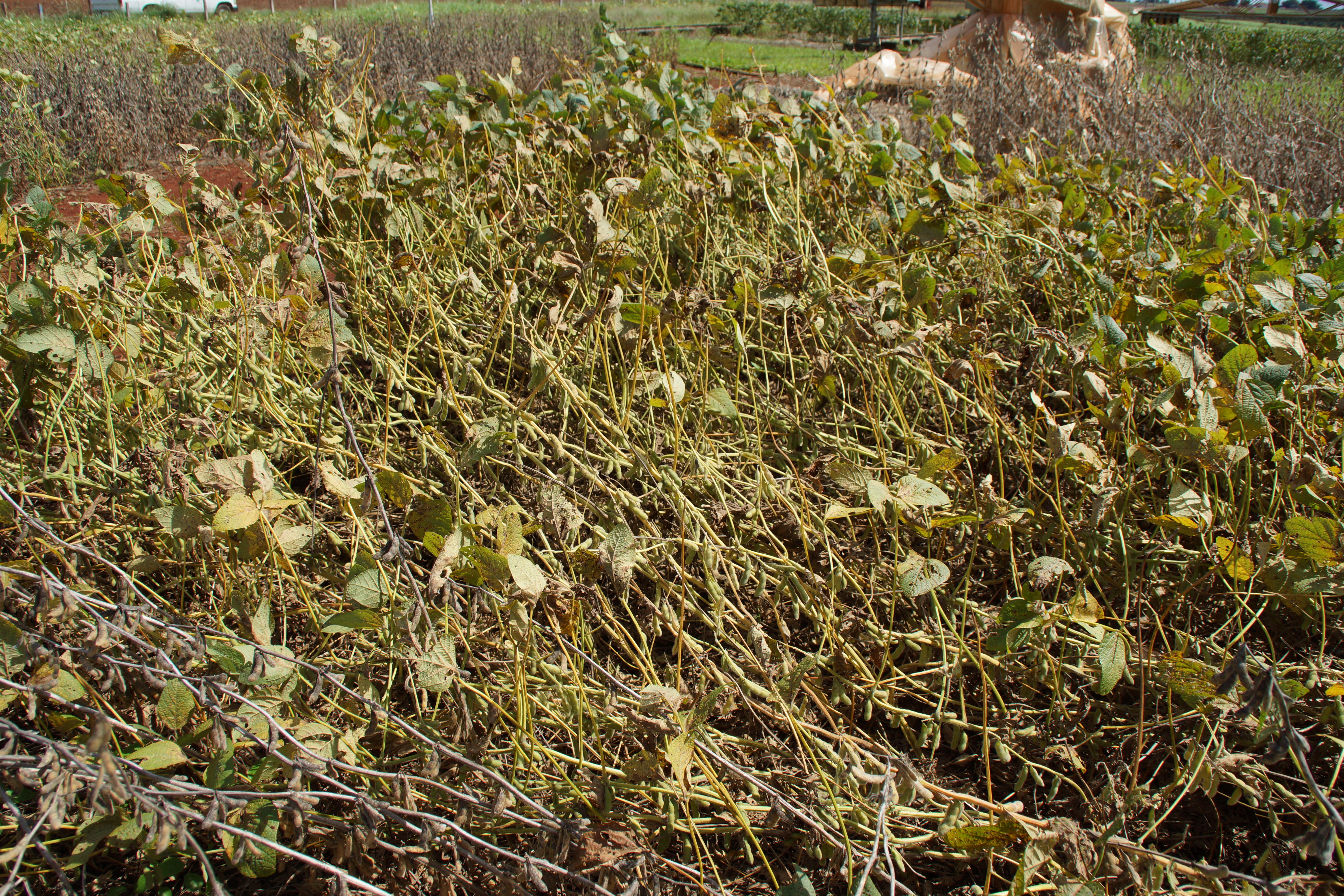
Additional File 2 | Photo from lodging and pod details from 1Ea2939 line
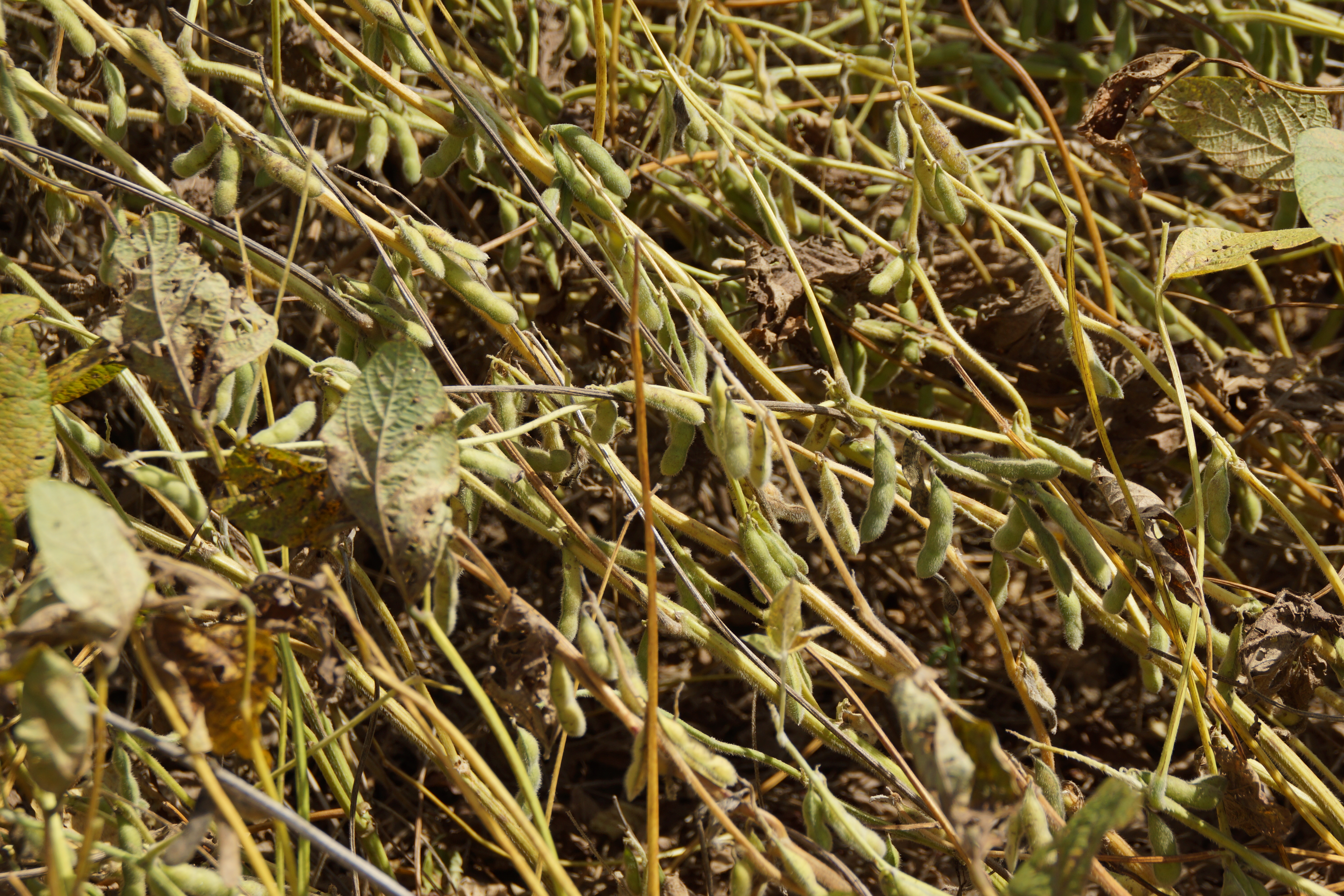


**A**

**B**

Supplement: FILE S2 — Photo from lodging and pod details from 1Ea2939 line. Lodging occurred after plentiful rain (341.4 mm) in a short period. [file Data_Sheet_2.DOCX]
