# Supplementary material for: Characterization of Soybean Genetically Modified for Drought Tolerance in Field Conditions
Source: Front Plant Sci. 2017 Apr 11;8:448. doi: 10.3389/fpls.2017.00448 (PMC5387084; doi:10.3389/fpls.2017.00448)
Supplement: FILE S3 — Climatologic water balance from crop season 2013/2014, showing rainfall (mm), water deficit, water withdrawal and maximum temperature (°C). Graphic is scaled in a 10-day period from October 2013 to April 2014. [file Data_Sheet_3.DOCX]

Additional file 3_Climatologic water balance_crop season_2013_2014
