# Supplementary material for: Characterization of Soybean Genetically Modified for Drought Tolerance in Field Conditions
Source: Front Plant Sci. 2017 Apr 11;8:448. doi: 10.3389/fpls.2017.00448 (PMC5387084; doi:10.3389/fpls.2017.00448)
Supplement: FILE S4 — Number of nodes and total pod number per plant for GM lines 1Ab58, 1Ea2939, and 1Bb2193 and WT plants (BR 16 cultivar) on February 17th, 2014 and on harvest (April, 2014), in non-irrigated (NIRR) and irrigated (IRR) treatments under field conditions. [file Data_Sheet_4.DOCX]

Additional file 4_Number of nodes and total pod number per plant
