# Supplementary material for: Characterization of Soybean Genetically Modified for Drought Tolerance in Field Conditions
Source: Front Plant Sci. 2017 Apr 11;8:448. doi: 10.3389/fpls.2017.00448 (PMC5387084; doi:10.3389/fpls.2017.00448)
Supplement: FILE S5 — Climatologic water balance from crop season 2014/2015, showing rainfall (mm), deficit, and water withdrawal, scaled in a 10-day period from October 2014 to April 2015. [file Data_Sheet_5.DOCX]

Additional file 5_Climatologic water balance_crop season_2014_2015
